# Strengthening and utilizing response groups for emergencies flagship: a narrative review of the roll out process and lessons from the first year of implementation

**DOI:** 10.3389/fpubh.2024.1405174

**Published:** 2024-05-15

**Authors:** Ishata Nannie M. Conteh, Fiona Braka, Edea Zewdu Assefa, Ebenezer Obi Daniel, Reuben Opara Ngofa, Joseph C. Okeibunor, Otto Emmanuel Omony, Jean Leonard Hakizimana, Alemu Wondimagegnehu, Mamoudou H. Djingarey, Aminata Grace Kobie, Doris Gatwiri Kirigia, Jerry-Jonas Mbasha, Senait Tekeste Fekadu, Olaolu Moses Aderinola, Adam Ahmat, James Avoka Asamani, Raymond Bernard Pallawo, Luigino Minikulu Mpia, Mor Diaw, Mamadou Kourouma, Kokou Davi, Siaka Condé, Kentse Moakofhi, Kumshida Yakubu Balami, Mie Okamura, Roselina Johanna De Wee, Gabriel Joseph, Grace Elizabeth Saguti, Ghirmay Redae Andemichael, Patrick Abok, Michael Avwerhota, Martins Chibueze Livinus, Henry Anayochukwu Okoronwanja, Lyndah Makayoto, Alfred Rutagengwa, Mawule Mady Ba, Youba Kandako, Pistis Manzila Livinus, Amadou Mouctar Diallo, Gervais Leon Folefack Tengomo, Marie Roseline Darnycka Belizaire, Arsène Daizo, Biranga Muzi, Abdoulaye Yam, Otim Patrick Cossy Ramadan, Lala Moulaty Moulaye D'khil, Boukare Bonkoungou, Helena O'malley, Abdou Salam Gueye

**Affiliations:** ^1^World Health Organization, Regional Office for Africa, Emergency Preparedness and Response Cluster, Brazzaville, Republic of Congo; ^2^World Health Organization, Regional Office for Africa, Emergency Preparedness and Response Hub, Nairobi, Kenya; ^3^Hubert Department of Global Health Rollins School of Public Health, Emory University, Atlanta, GA, United States; ^4^World Health Organization, Regional Office for Africa, Universal Health Promotion and Social Determinant, Brazzaville, Republic of Congo; ^5^World Health Organization, Regional Office for Africa, Universal Life Course, Workforce, Brazzaville, Republic of Congo; ^6^World Health Organization, Country Office, Nouakchott, Mauritania; ^7^World Health Organization, Country Office, Niamey, Niger; ^8^World Health Organization, Country Office, Lome, Togo; ^9^World Health Organization, Country Office, Gaborone, Botswana; ^10^World Health Organization, Country Office, Abuja, Nigeria; ^11^World Health Organization, Country Office, Windhoek, Namibia; ^12^World Health Organization, Country Office, Dar es Salaam, Tanzania; ^13^World Health Organization, Country Office, Addis Ababa, Ethiopia; ^14^World Health Organization, Country Office, Nairobi, Kenya; ^15^World Health Organization, Country Office, Kigali, Rwanda; ^16^World Health Organization, Country Office, Dakar, Senegal; ^17^World Health Organization, Country Office, Brazzaville, Republic of Congo; ^18^World Health Organization, Country Office, Kinshasa, Democratic Republic of Congo; ^19^World Health Organization, Country Office, Bangui, Central African Republic; ^20^World Health Organization, Country Office, Ndjamena, Chad

**Keywords:** SURGE, emergencies, public health, response, African region

## Abstract

The World Health Organization Regional Office for Africa (WHO/AFRO) faces members who encounter annual disease epidemics and natural disasters that necessitate immediate deployment and a trained health workforce to respond. The gaps in this regard, further exposed by the COVID-19 pandemic, led to conceptualizing the Strengthening and Utilizing Response Group for Emergencies (SURGE) flagship in 2021. This study aimed to present the experience of the WHO/AFRO in the stepwise roll-out process and the outcome, as well as to elucidate the lessons learned across the pilot countries throughout the first year of implementation. The details of the roll-out process and outcome were obtained through information and data extraction from planning and operational documents, while further anonymized feedback on various thematic areas was received from stakeholders through key informant interviews with 60 core actors using open-ended questionnaires. In total, 15 out of the 47 countries in WHO/AFRO are currently implementing the initiative, with a total of 1,278 trained and validated African Volunteers Health Corps-Strengthening and Utilizing Response Groups for Emergencies (AVoHC-SURGE) members in the first year. The Democratic Republic of Congo (DRC) has the highest number (214) of trained AVoHC-SURGE members. The high level of advocacy, the multi-sectoral-disciplinary approach in the selection process, the adoption of the one-health approach, and the uniqueness of the training methodology are among the best practices applauded by the respondents. At the same time, financial constraints were the most reported challenge, with ongoing strategies to resolve them as required. Six countries, namely Botswana, Mauritania, Niger, Rwanda, Tanzania, and Togo, have started benefiting from their trained AVoHC-SURGE members locally, while responders from Botswana and Rwanda were deployed internationally to curtail the recent outbreaks of cholera in Malawi and Kenya.

## 1 Introduction

The public health emergency challenge in the African region, with an estimated 100 occurrences annually, has been enormous and of divergent nature, with consequences associated with multiple episodes of emerging and re-emerging diseases, including disasters due to natural hazards such as earthquakes, droughts, floods, and storms (1). The weakness of the health system and its inherent pillars—governance, human resources for health (HRH), health information management, service delivery, medicines, related health product supply, and financing have been established in the African region (2, 3). The emergence of COVID-19 further revealed the weak status of the health system in Africa, notably inadequate preparedness for the pandemic resulting in substantial impact (4–6). It was further exposed that the response to the pandemic in the WHO/AFRO was considerably fair but was not in tandem with the recorded magnitude (7). Scientists suggest the need for the African region to enact interventions that will give the African region’s health system’s strength, resilience, and financial capacity a face-lift using a contextualized methodology (8).

Before the pandemic, the WHO/AFRO member states experienced multiple emergencies—diseases and disasters due to natural hazards with multi-faceted consequences and severe pressure on the health system. Among the many epidemics, a few are worth mentioning because of the scale, the impact on the health systems, and the associated economic losses within the region. One such very conspicuous infectious disease outbreak is the Ebola virus disease (EVD), a fatal hemorrhagic disease that affects both humans and primates. It was first discovered in Sudan and the Democratic Republic of the Congo (formerly Zaire) in 1976 and ravaged many African countries afterward (9). Outbreaks of EVD have been recorded in multiple countries, including Gabon, Uganda, Guinea, Sudan, Liberia, Sierra Leone, and the Republic of Congo (9). The disease recorded the highest impact between 2014 and 2016 in West African countries, with unprecedented emergency response and considerably high mortality (9, 10).

Similarly, the cholera outbreak in the African region has been a recurrent event with multiple responses before the COVID-19 pandemic (11). Globally, no fewer than 4 million cases and 143,000 mortalities are associated with cholera annually, out of which 54% emanate from Africa, with evidence of recent outbreaks in some countries, including Malawi, Ethiopia, Kenya, Zambia, Zimbabwe, and South Africa (11–14). The additional emergency burden contributed by climate change and disasters due to natural hazards in the African region is also a factor to consider. In the last two decades, the frequency of disasters due to natural occurrences with devastating impacts in Africa has been unprecedented (15). Typically, the occurrence of Tropical Cyclone Freddy in Malawi across many districts in 2023 amidst the response to the most impacting cholera outbreak resulted in compounded effects on the inhabitants and, most significantly, the vulnerable population—women and children, with multi-faceted humanitarian needs (16). By extension, climate change and disasters due to natural hazards have been identified as banes to agricultural development and advancement in the African region. Consequently, these create a challenge for the continuous availability of one of the most crucial human needs, with serious consequences for achieving the related Sustainable Development Goals (SDGs) (17–19).

Researchers have argued that the experience of the African region with the COVID-19 pandemic was associated with unforeseen negative consequences of morbidities and mortalities in the human race. It has been revealed that the health systems in the African region need overhauling interventions to be more adequately prepared for future emergencies of great magnitude without disrupting the provision of essential health services (20, 21). However, it is indispensably clear that HRH, along with its effective management, stands out among the health system pillars, and its importance in responding efficiently and effectively to emergencies cannot be overemphasized. This is because it drives other health system pillars into full operational functions and translates the public health inputs into the desired outputs, outcomes, and impacts (22). The lessons learned from the global impacts of the pandemic on trained public health responders, as well as the attendant decline in their numbers across the continent of Africa at a time when their services are most required, highlight the importance of giving this health system pillar the attention it requires, including training and re-training geared toward improving the emergency management system in the African region (23, 24).

In addition to this, critical recommendations that were made at the global level, including those by the Independent Panel on Pandemic Preparedness and Responses (IPPPRs), the International Health Regulations 2005 (IHR) Review Committee, the Independent Oversight and Advisory Committee for the WHO Health Emergencies Programme (IOAC), the 74th World Health Assembly (WHA74), and the G7 and G20 Health Declarations, need to be translated into African-driven solutions that reflect regional, national, and local needs.

Against this backdrop, there was a need for improved health security in the African Region; thus, the WHO/AFRO, in collaboration with the Africa CDC, launched the emergency preparedness and response (EPR) flagship initiative in 2022 to enhance the capacity of all member states to prepare for, detect, and respond to public health emergencies in line with the relevant tenets of the World Health Organization (WHO) (25). The EPR flagship initiatives include Promoting Resilience of Systems for Emergencies (PROSE); Transforming African Surveillance Systems (TASS); and—Strengthening and Utilizing Response Groups for Emergencies (SURGE). SURGE was recognized as an indispensable factor in achieving other initiatives and, hence, was prioritized in WHO/AFRO to build on the existing structure toward strengthening the capacities of the African emergency management systems (25).

This article was prepared to document the stepwise process involved in rolling out the SURGE flagship initiative within the first year using the available and verifiable planning and operational documents, including triangulating the lessons learned across the first batch of implementing countries through key informant interviews (KIIs) conducted after the1-year implementation of the SURGE flagship initiative. It was opined that the KII would facilitate helpful feedback from the stakeholders who actively participated in the roll-out process. The focused thematic areas include the roll-out facilitating factors, best practices, challenges faced during implementation and how they were addressed, and suggested recommendations for future improvement.

## 2 Methodology

### 2.1 Study design

This was a descriptive survey design with qualitative approaches to collect critical data and information.

### 2.2 Study participants and sampling

The targeted participants in this study were AVoHC-SURGE initiative core actors at the WHO Country Office (WCO), and the Ministry of Health (MoH) across 15 countries where the initiative was implemented in the first year. This was aimed at generating an in-depth understanding of the initiative’s operations from participants with rich information rather than engaging a representative sample of all stakeholders involved in the implementation. The main criterion for participation was the involvement in the implementation of the initiative. The team leads at the WHO and MoH were requested to nominate members with core knowledge of the teams’ participation from inception to date. The confidentiality and anonymity of the participants were maintained, as no demographic information was shared.

### 2.3 Document reviews

We identified and utilized documents pertaining to the AVoHC-SURGE planning and implementation at WHO and MoH with a specific search for information on the initiative’s activities. Those documents include planning and operational reports from the WHO and MoH, previous update meetings, oversight board meetings from the central repository of the WHO, and record notes. For quality improvement purposes, the review team conducted a point-based search for elements around the focus of the study.

### 2.4 Key informant interview

Information was collected from purposively identified key informants. They included planning implementation committee members (15), implementation country coordinators (15), and 2 core AVoHC-SURGE field supervisors from each of the 15 countries (*n* = 30). A request for information was sent to the participants to schedule an in-depth interview at their convenience while two trained research assistants conducted a face-to-face interview with each of them, which took between 30 and 45 min. For those who were unable to participate due to exigent reasons, they were requested to designate an alternative participant to represent them. Further provision was made for those who could still not make it by providing them with a list of questions requesting their written feedback.

### 2.5 Data analysis

Verbatim transcription of all audio files was performed by the WHO AFRO TIP team. NVIVO software was used for effective organization during analysis, and we reviewed all transcripts for transcription accuracy.

A comparison with the audio files was performed, and data cleaning was conducted where required. We engaged in deep data familiarization, thematically generated related codes, grouped similar codes as required, and checked coherence between themes with the application of approved themes across the data before generating the findings. All authors reviewed and approved the findings.

### 2.6 Ethical considerations

Approval to conduct the study was obtained from the WHO-AFRO publication review committee. Participants gave their consent to the study while their anonymity, privacy, and confidentiality were ensured.

## 3 SURGE flagship initiative: the roll-out process

### 3.1 Scoping missions

Being a unique venture with a high degree of importance, the SURGE flagship initiative demanded an inclusive stakeholder partnership, participation, buy-in, and unwavering commitment. Scoping mission planning and execution was the first significant step of the initiative. Researchers have documented the benefits of this type of approach in public health project planning and implementation (26, 27). The importance of creating an atmosphere that will enable all public health and possibly non-public health partners in WHO/AFRO to accept the initiative as an all-important one, especially in these critical moments when the region continuously experiences multiple emergencies, is of paramount value. This will contribute to the region’s effort toward living up to the expectations of the IHR 2005 (28) and in tandem with the emergency response framework (ERF) (29)_._

**Figure 1:**
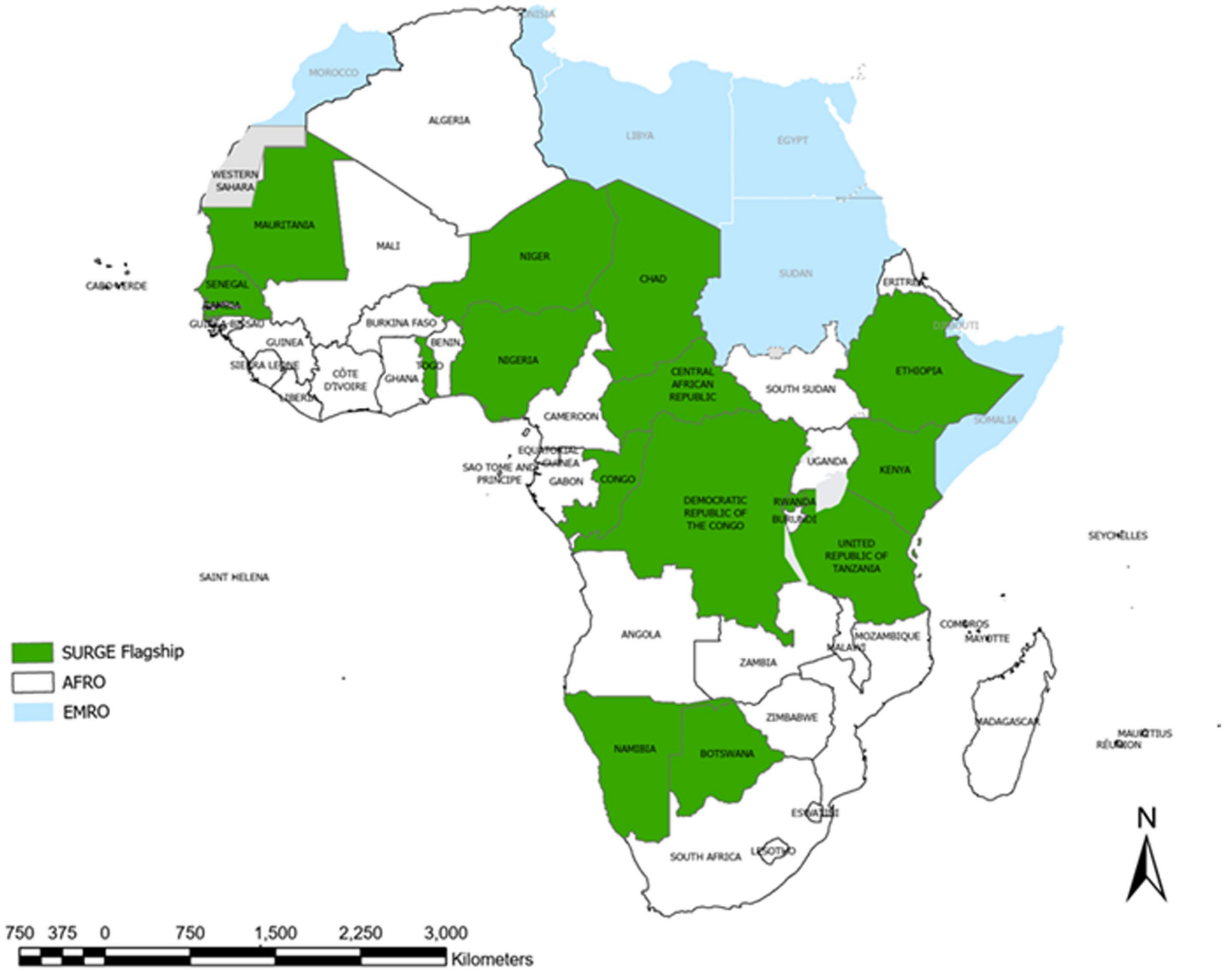
SURGE flagship initiative implementing countries across WHO/AFRO, 2022.

The scoping mission entailed the invitation through official communication to all stakeholders in the implementing countries for an initial orientation on the SURGE flagship initiative, a step that all other processes hinged on for a smooth rollout of the initiative. It involved high-level consultation of multifaceted/multi-functional government organizations, international non-governmental organizations, community-based organizations, local and international donors/development partners, and all other stakeholders that can propel the vision as applicable to each country with the expectation of facilitating good partners’ onboarding experiences and untoward actions that will lead to desired results (30). This critical step is well incorporated in the SURGE Implementation Monitoring Interactive Dashboard for the ease of status follow-up for each country (31) (see Figure 1).

Further to the above step, there was a holistic technical approach during the scoping mission toward the development of the SURGE flagship initiative Roadmap with the four pillars: workforce development, response readiness and coordination, operations and logistical support (OSL), and risk communications and community engagement (RCCE). With guidance from the technical mission team, the multi-sectoral technical in-country team was tasked with developing a costed roadmap in a workshop setting. The mission team facilitated the workshop, and the multi-sectoral country team conducted the gap and needs analysis, reviewing all existing and available documents and plans, including but not limited to the National Action Plan for Health Security (NAPHS) and EPR Plans. The team also identified the existing supporting structures within the countries and agreed on top priorities to address the gaps as per response to emergencies, paying keen attention to the four pillars of the SURGE flagship initiative. This action was corroborated by the relevant guidance documents of WHO and other partners, which stipulate the need for and benefits of gaps and needs assessment in emergency response (32–34). This resultantly led to all stakeholders’ efforts toward preparing a country-specific *2-year road map* complemented by a well-articulated work plan as a key deliverable of the scoping mission for each country in implementing the SURGE flagship initiative.

The road map was expected to serve as the pivot for action and the compass for direction in the whole process, with close monitoring of the progress through the designated interactive dashboard with an online roadmap tracker with a SharePoint accessible to all countries and updated regularly (31). This action toward preparing a country-specific roadmap through the scoping mission for the SURGE flagship initiative is in close tandem with and supports similar global action toward EPR, with the involvement of world leaders and global partners (35). On the last day of the scoping mission, a presentation of the draft road map was conducted to the Ministers and senior officials of the Government on the findings, with a focus on existing capacities, gaps, priorities, and activities developed into a draft costed road map. The estimated draft budget and next steps are presented, with a focal point appointed at the MOH and WHO to finalize the road map and implement activities. The scoping mission started in February 2022 and was completed in 15 countries, with Mauritania being the first and Ethiopia the last country to achieve this level in November 2022.

### 3.2 Post-scoping mission action

At this stage, the roadmap was subject to a comprehensive review with sectional-based analysis to revamp the content to select and re-organize the set ambitious goals for more innovative objectives/goals. The activities were budgeted as appropriate, with deadlines set for each activity. The relevance of setting SMART goals, along with its benefits in achieving the desired results in health projects, has been well-established by researchers (36, 37). At this critical stage, the technical team called for another meeting of all stakeholders (those who attended the scoping missions) to present the costed road map for the stakeholders’ comprehensive review, with further recommendations and ensuring that all the stakeholders agree to the content as acceptable and operable. This is referred to as ‘road map validation.’ This is conducted with relevant WHO and partners’ procedures and guidance, which emphasizes this procedure before implementing public health projects (38). The member states’ leadership endorsed the roadmap for government ownership and leadership for implementation. After endorsement, the road map became an official document for the SURGE flagship initiative implementation in each of the implementing countries for 2 years and is closely monitored through an online dashboard (31).

### 3.3 Establishment of in-country multi-sectoral and multidisciplinary AVoHC-SURGE team

The member states established multi-sectoral selection committees from relevant sectors under the leadership of the MoH and/or National Public Health Institute to manage multi-sectoral and multidisciplinary response teams. The selection committee was guided by well-laid-out terms of reference (ToR) and the WHO Global Strategy on HRH. To enhance a broader pool for robust selection, the committee in each country mapped and identified multi-sectoral and multidisciplinary teams comprising 50-214 experts from relevant government sectors following the One Health approach (39) to serve as AVoHC-SURGE members empowered by participating in the mandatory onboarding and specialized training. The profiles of the AVoHC-SURGE members include but are not limited to epidemiologists, laboratory experts, anthropologists, entomologists, veterinarians, data managers, field logistics and operation, infection prevention and control, risk communication and community engagement (RCCE), gender-based violence, water sanitation and hygiene, nutrition, finance and administration, mental health and psychosocial support, incident manager, case management, vaccination, planning/monitoring, and evaluation.

The importance and benefits of adopting a multi-sectoral/multidisciplinary approach in public health management have been highlighted (40). This is in tandem with the main objective of SURGE flagship initiative, which is to produce at least 3,000 African core responders of diverse expertise ready to be deployed within the first 24–48 h of an emergency. This is achieved through strengthened workforce development, enhanced response readiness and coordination mechanisms, timely and effective operational and logistics support, and enhanced RCCE. The approach ultimately addresses the quest to align more efficiently with the relevant emergency framework (29) and reduce the existing gap in the efficacy of emergency response within the WHO/AFRO, as echoed by the public health stakeholders in the region (41–43).

In a more granular form, the SURGE flagship initiative envisioned a safer African region where outbreaks and other health emergencies have limited morbidity, mortality, and socioeconomic disruptions, with the following key objectives, which all countries are focused on achieving:

- *Workforce development* aims to enable the fast mobilization of high-caliber African responders to shorten the response time to emergencies, with the target of effective deployment within the first 24–48 h.
- *Response readiness and coordination* aim at establishing Public Health Emergency Operations Centers (PHEOCs) as a unique coordination point for managing all EPR activities as recommended by the IHR (2005) (28).
- *Operations support and logistics* aim to facilitate the prompt and effective deployment of emergency supplies.
- *RCCE aims* to create a robust and inclusive information dissemination structure around public health emergencies.

### 3.4 In-country training and central rostering

Prior to the face-to-face training, the participants completed some mandatory preliminary online WHO and relevant training to prepare the ground for a smooth knowledge acquisition kick-off. With support from the WHO/AFRO, in collaboration with the WCO and the MoH focal points, the training team conducted face-to-face onboarding training for the selected multidisciplinary AVoHC-SURGE members in emergency management skills. The training included Public Health Emergency and Operation Centre (PHEOC) and Incident Management System (IMS), Humanitarian Cluster Coordination, Rapid Response Team, Gender-based violence, Preventing and Responding to Sexual Exploitation, Abuse and Harassment, and External Communication. This was then followed by specialized training in specific technical areas such as case management, infection prevention and control, surveillance, and laboratory. The training was carried out with close attention to the relevant WHO guidance on the national response to health emergencies and disasters (32), the expected quality level of training for health emergency workers, and the global strategy on HRH (25).

Different scenarios or approaches were used to implement the face-to-face onboarding AVoHC-SURGE members training. The scenario to be used was discussed and agreed between the country and WHO based on the country’s context, specificities, number of trainees, and facilitators.

The approaches include the following:

- *Back-to-back*: All four modules of the onboarding training were implemented in a single block without a break in between; in this case, the AVoHC-SURGE members were trained together.
- *Waves:* The AVoHC-SURGE team was divided into cohorts and then trained using the back-to-back approach.
- *Checkerboard:* All the AVoHC-SURGE team was trained together, module by module but with a break period between modules.
- A combination of two or more of the above.

To ensure the quality of the training, a series of facilitators’ preparatory meetings, both virtual and face-to-face, were conducted to review and adapt training materials according to the country’s context; participants did pre- and post-tests, daily evaluation of the training and feedback from participants, as well as facilitators daily meetings to assess what went well, what did not work well, and the plan of improvement in the subsequent sessions. The trained AVoHC-SURGE responders were rostered into the WHO/AFRO database for ease of deployment at national and international levels. In collaboration with the country offices, WHO/AFRO equipped the responders and country offices with necessary safety and visibility kits and logistics and supplies, including the provision of field vehicles comprising ambulances, pick-up vans, and sports utility vehicles to facilitate timely response to public health emergencies in each country. WHO/AFRO monitors and evaluates the implementation progress and outcomes to ensure accountability, drive progress, and create learning opportunities. It also ensures the collection of feedback from training sessions, holds bi-weekly meetings with focal points for periodic updates, develops and implements progress tracking tools, and tracks key performance indicators (KPIs) in quarterly reports, as evident in the designated dashboard (31) (see Table 1).

**Table 1:** Modules, course component/package, and the duration of AVoHC-SURGE training.

Module	Course component/ package	Duration (Days)
Module 1	Public Health Emergency Operation Centre, Incident Management System, and Emergency Response Framework.	7
Module 2	Humanitarian Overview and Health Cluster Coordination	5
Module 3	Rapid Response Teams (RRTs), including Infection Prevention and Control (IPC), Laboratory and Media, and External Communications;	9
Module 4:	Gender-Based Violence (GBV) and Prevention and Response to Sexual Exploitation, Abuse, and Harassment (PSEAH).	3

## 4 Outputs of the initiative in 1 year of implementation

### 4.1 Consultation of high-level government officials

**Figure 2:**
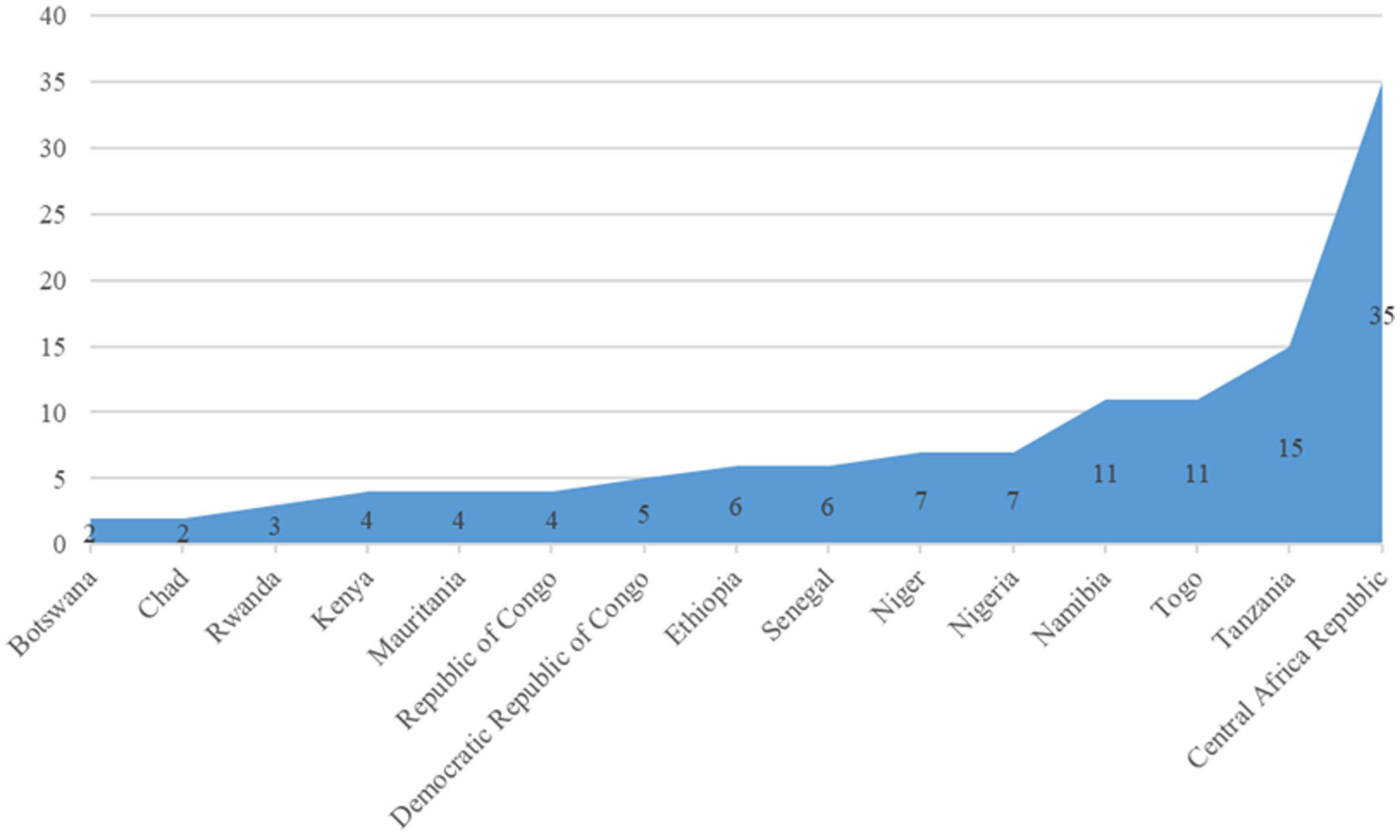
Distribution of the high-level government officials consulted during the scoping mission.

The scoping mission team consulted 122 high-level government officials (that included health ministers, permanent secretaries, and directors) in 15 countries, with the resultant achievement of the drafting of a 2-year roadmap with all stakeholders along with the Memorandum of Understanding (MoU) to ensure the commitment of WHO and government in the implementation of the SURGE flagship initiative (see Figure 2).

The probable determining factor for the number of high-level government officials consulted in each country during the scoping mission is multifaceted in nature. These may include the number of officials responsible for the office functions, availability of the concerned officials hinged on other functions, exigent functional role designations from higher authority, pre-designed organogram within the government structure, and other miscellaneous factors, as experienced in the process. They are the representatives of the member states who facilitated the finalization, validation, and endorsement of the roadmap for the implementation of the SURGE flagship initiative in all 15 countries.

### 4.2 Financial implications

**Figure 3:**
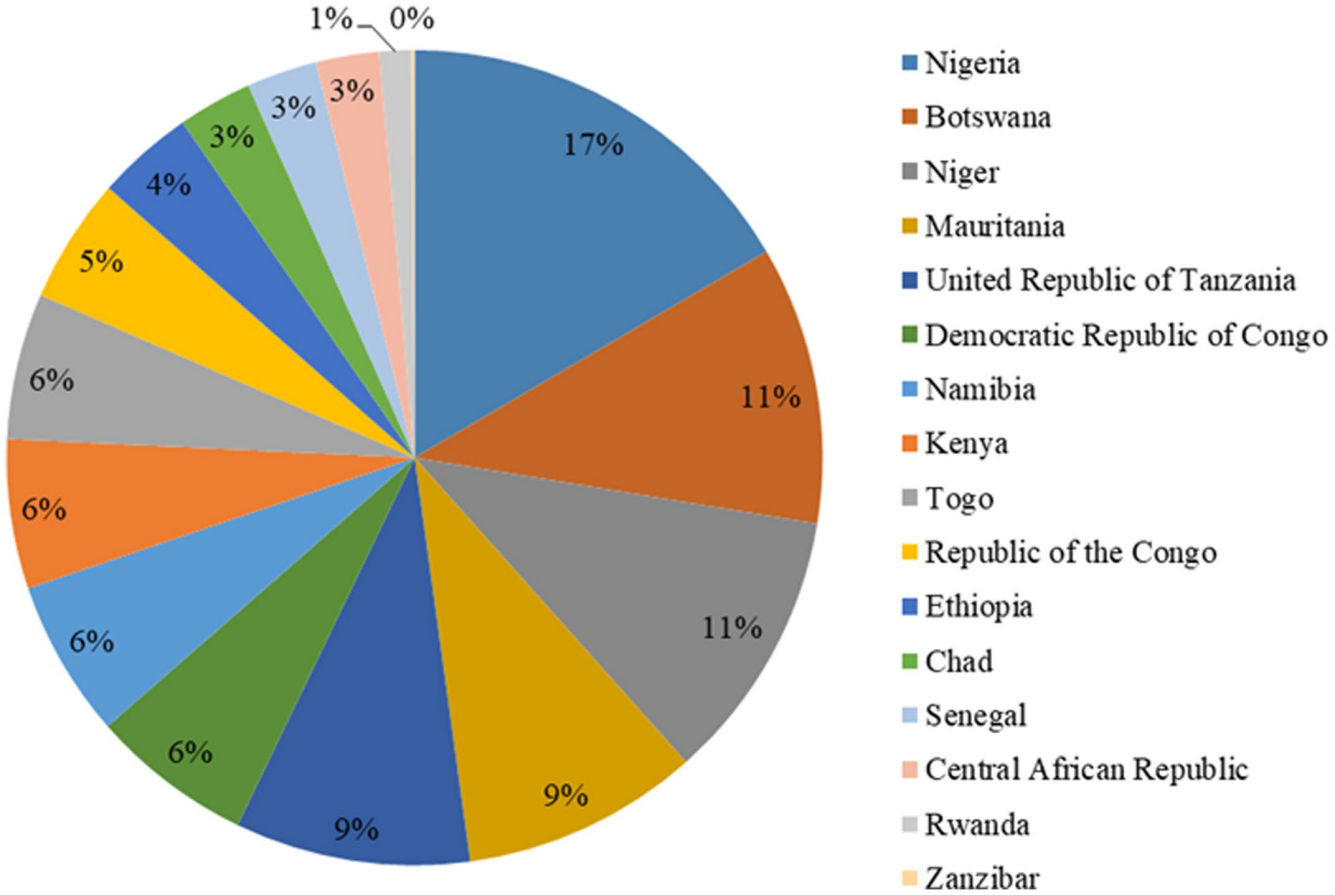
Proportional distribution of financial support from WHO/AFRO.

The implementing countries estimated the requirement of *USD 326,239,011.9* budget to implement the flagship, with the operational support and logistics pillar covering over half of the entire budget (55%), followed by the response readiness and coordination pillar (20%) and workforce development pillar (18%). WHO/AFRO has supported countries in the initiation of implementation of the SURGE initiative with USD *9,061,228* with Nigeria having the highest proportion (17%) of the financial support (31) (see Figure 3).

**Figure 4:**
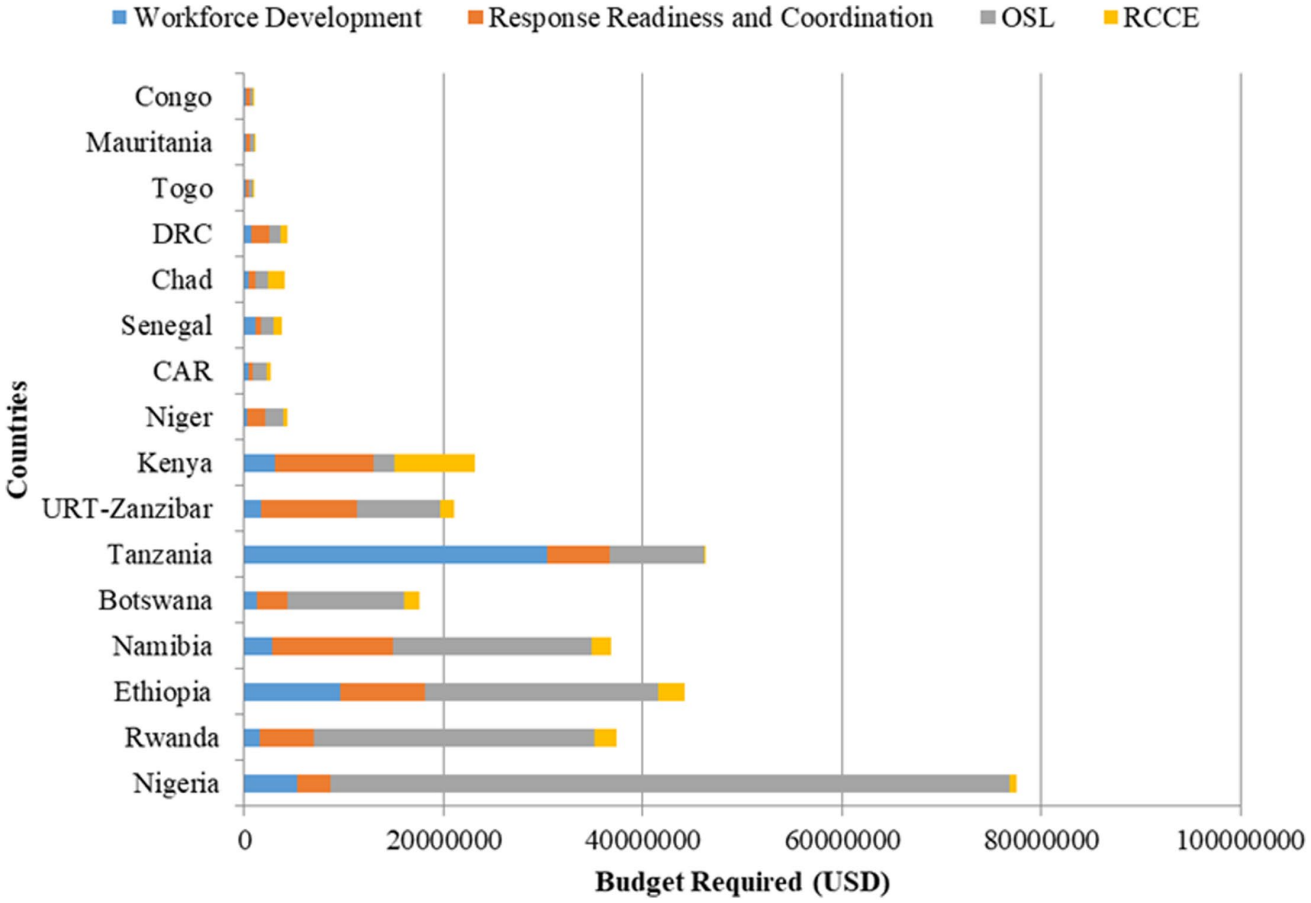
Distribution of countries SURGE flagship initiative roadmap budget requirements.

A signed MoU by the member states serves as a tool for resource mobilization from all relevant stakeholders and partners to support the implementation of the roadmap activities. This was expected to serve as the primary source of the financial propeller for the implementation of the initiative. Katz et al. unveiled different sources of funding for health financing, with the most recurrent being country budgets and risk pooling (44). However, the World Bank and other stakeholders recognized that most African countries’ budget for health financing is still far lower than that of other countries in the developed world, with most countries in WHO/AFRO expending less than 5% of their annual budget for health financing (45, 46). This necessitates collaborative donations for health financing across African countries as advised by WHO, World Bank, and Global Fund (47), allowing WHO to continuously play its normative roles in the world of public health (48). Hence, the approach of multi-stakeholder sourcing for financing the SURGE flagship initiative across the implementing countries is technically hinged on these facts to ensure sustainable financing of the initiative until the desired goal is achieved. Nigeria has the highest roadmap total budget requirements of *77.5 million*. The detailed distribution is shown in Figure 4.

### 4.3 Training outputs

**Figure 5:**
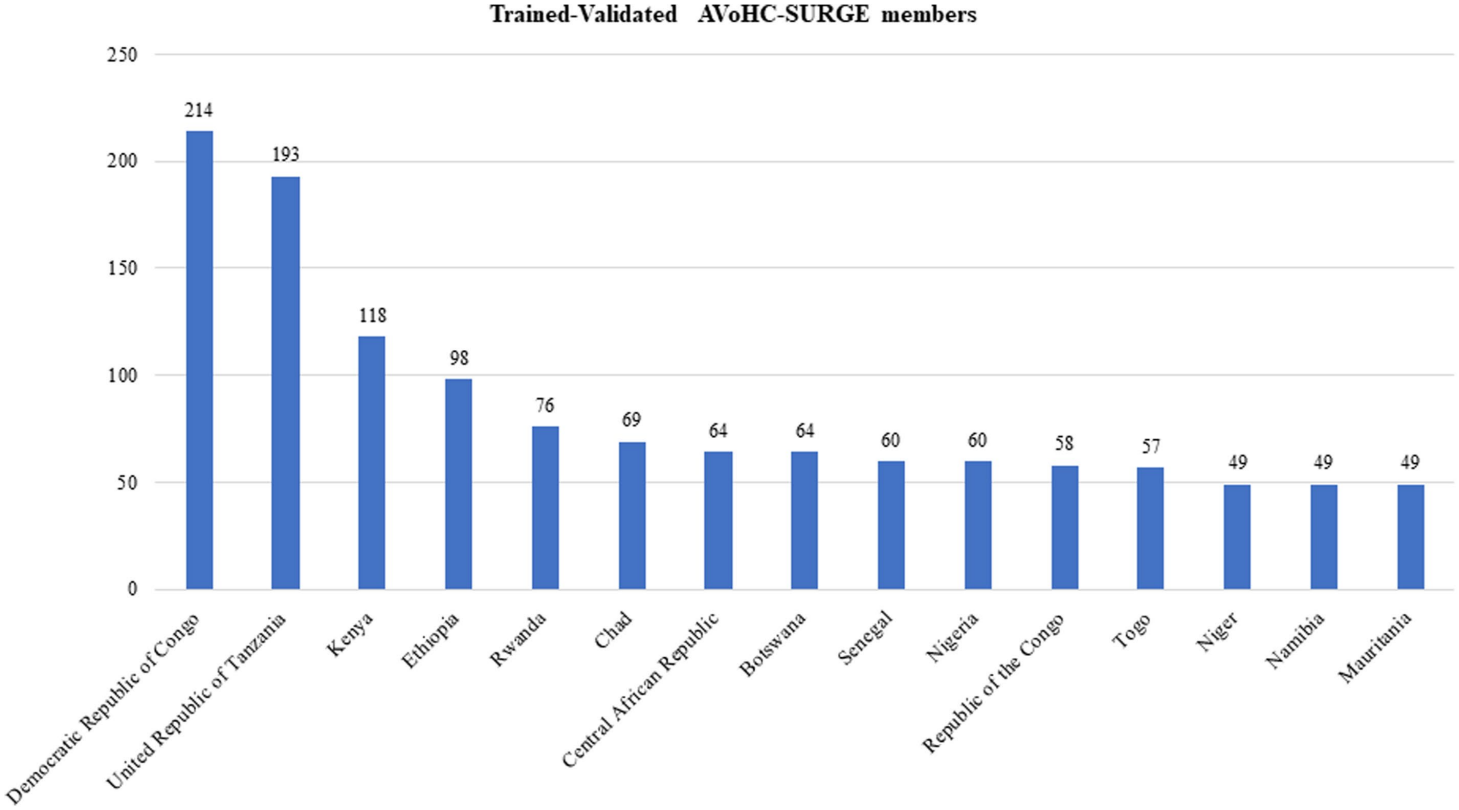
Bar chart showing the distribution of AVoHC-SURGE members trained by onboarding training modules.

The training outputs in the implementing countries are diverse and are represented in Figure 5. The minimum number of trained-validated SURGE members expected from each country as targeted from inception is 50.

As of the study period, the total number of trained-validated AVoHC-SURGE members across all 15 countries is *1,278*; of which, 214 were from the Democratic Republic of Congo (DRC), the country with the highest proportion of trained SURGE members (16.7%). At the same time, Niger, Namibia, and Mauritania have equal and the lowest number (48), each representing 3.8% of the total number of trained AVoHC-SURGE members. In total, 12 out of the 15 implementing countries (80%) have achieved the cutoff point of a minimum of 50 trained-validated AVoHC-SURGE members expected from each country. It is noteworthy to state that the output of trained AVoHC-SURGE members from Tanzania (193) is a combined achievement from the mainland (137) and Zanzibar (56).

The DRC, with the lowest budget (approximately USD 997,000) and 6.4% of the proportion of financial support from the WHO/AFRO (31), recorded the highest number of trained AVoHC-SURGE members to date, while Nigeria, with the highest budget (USD 77.5 million) and the highest proportion of financial support from WHO/AFRO (17%) (31), is among the countries with the lowest numbers of trained AVoHC-SURGE members within the last one year of implementing the initiative. It is noteworthy to state that many determining factors may be responsible for the number of trained AVoHC-SURGE members (government policies, technical, logistics, etc.); the output in this regard should be interpreted with caution, but the notable success recorded in the DRC might have been technically connected with, among other factors, the adoption of the principle of efficient project finance management, which Monteiro et al. associated its efficacy with the active and unwavering participation of the project management office (PMO) and critical actors’ effectiveness for maximum output (49).

The relatively low number (60) of the validated trained AVoHC-SURGE members in Nigeria and other low numbers in a few other countries, which is the most important output of the initiative, seems to be uncorrelated with the objectives of the NAPHS of Nigeria (for example), which posited that there are multiple annually reported diseases epidemics and other public health threats in the country which has the highest population on African continent (50), supported by the WHO strategy (2022–2026) for the NAPHS (51). While this output may not be finite (as figures are as at the study period) and the initiative has not yet ended, it may, however, not be incorrect to state that this direction of thought may also apply to other countries with considerably low numbers of trained AVoHC-SURGE members and who are in similar health security situations. This implies that the current milestone reached by some WHO/AFRO member states that are currently implementing the SURGE flagship initiative, though appreciable, may require an increased level of commitment from all stakeholders piloted by the member states’ governments toward increasing the number of trained AVoHC-SURGE members/responders for emergency events.

### 4.4 Immediate benefits of the SURGE flagship initiative

As a positive ripple effect of the SURGE flagship initiative, six countries, namely, *Botswana, Mauritania, Niger, Rwanda, Tanzania, and Togo*, have benefited from their trained AVoHC-SURGE members locally by deploying emergency responders/experts to detect, assess, and respond to polio, rift valley fever, yellow fever, cholera, measles, malaria, meningitis, diphtheria, Marburg virus diseases outbreaks, flooding, and inundations in their countries (52, 53). Furthermore, member states have started benefiting from the international deployment of the trained AVoHC-SURGE members through the WHO Regional Office for Africa coordination to facilitate immediate responses to the cholera outbreak in Malawi and Kenya by deploying AVoHC-SURGE responders from Botswana and Rwanda. To ensure improved effectiveness and efficiency in the system regarding further deployment and better management of the WHO/AFRO emergency workforce, the organization is arranging a deployment approach through the Global Outbreak Alert and Response Network (GOARN) (54).

### 4.5 Feedback from the key actors on the roll-out process

The following thematic areas highlight the feedback from the key actors/respondents on the roll-out process of the initiative:

#### 4.5.1 Roll-out facilitating factors

The responders reported a good number of factors that have facilitated the roll-out of the SURGE flagship initiative in the currently implementing countries, and they are either justifications for or propellers of the initiative. As highlighted by the responders, these facilitating factors are centered on the existing low health system efficacies, exposure to multiple emergencies, the uniqueness of the approach, and the monitoring mechanism set in motion, among others. The excerpts from a few respondents are as stated as follows:

*“This is a big country with complex emergencies (conflict, drought, other natural disasters, and multiple disease outbreaks), a fragile health system, and limited capacity to respond to all these emergencies, though with the appreciable existing structure to kick off, justified the initiation and rollout of SURGE in this country.”* **Country SURGE flagship initiative focal person**

*“The existence of the multi-sectoral selection committee, the WHO country office teamwork with the government, the involvement and commitment of the Ministry of Health, and the implementation of a one-health approach when recruiting experts, were all instrumental in facilitating a robust roll-out.”* **One Health Committee member**

*“The regional office closely monitored the implementation and roll-out using an online dashboard, and monthly calls were held to provide general information and get updates from the country offices on the progress made on implementing the country roadmap. Funds mobilized for the initiative were disbursed by the regional office for the implementation of the roadmap in the countries.”* **Regional Coordinator**

#### 4.5.2 Best practices

Most respondents applauded some key best practices, including, among others, the involvement of multiple government and private structures, the unique training methodology, and exposure to the simulation exercise (SimEx), which are conspicuously perceived as best practices. This is well corroborated in the key informants’ responses, as quoted below:

*“I find an aspect very innovatively thoughtful in the process, which I obviously considered as one of the best practices, and that is the involvement of decentralized administrative structures (regional governors, regional health departments, etc.) and the involvement of the medical association, through which the private sector can be brought on board.”* **MoH Focal Person**

*“The multi-sectoral and multi-disciplinary aspect of selecting the national SURGE team members and the training methodology that includes practical exercises, such as tabletop exercises, well garnished with simulation exercises, are all notable best practices in the implementation of the SURGE initiative.”* **External Relations and Partnerships Coordinator**

#### 4.5.3 Challenges and circumventing strategies

The most frequently observed challenge of the initiative during the 1-year implementation is inadequate finance. Most respondents stated this as the most daunting hurdle some stakeholders are presently strategizing to overcome through country-specific approaches. Selected responses from the field corroborate this.

*“Outside of training, the SURGE coordination no longer had the means to continue the activities contained in the roadmap. Currently, we have difficulties with financial resources to implement the other SURGE activities including specific training. We hope to address this as we are currently consulting with potential donors to have headway.”* **MoH Focal person**

*“We had a delay in the approval of the EPR flagship roadmap and also in identifying SURGE trainees by regional health bureaus along with an inadequate budget to train more than 100 as anticipated. We are, however, able to conduct advocacy visits to the public health institute and MoH leadership and conduct weekly meetings with EPR flagship members in-country to resolve some of the challenges.”* **Country SURGE flagship initiative coordinator**

### 4.6 Lessons learned

The key informants responded directly to highlighting lessons learned throughout the process. Governments’ actions in taking leadership responsibilities with resultant ownership, the importance of all stakeholders’ cooperation in achieving health project objectives, and the essentiality of kick-off financial support in implementing health initiatives with associated encouragement are among the lessons that were reportedly learned in the SURGE initiative rollout in the last year.

*One good lesson learned is that initiatives of this kind may be difficult to implement without the commitment of all parties to the policy. In addition, the Sectoral Ministries have given a significant boost to this initiative by providing full latitude to experts from their departments to take part in the* var*ious training courses.* **Selection Committee Member**

*“It is good to learn that advocacy with senior managers (ministers) facilitates collaboration and understanding in planning and implementation and that the timely availability of kick-off funds facilitates encouragement towards early implementation of activities. I also like to say that planning and logistics are key, and early communication and release of letters are ‘catalytic agents’ for attendance and acceptance.”* **EPR Flagship Coordinator**

*“There are benefits associated with ensuring that the government leads the entire process and that other partners’ roles are more of technical guidance. Also, while seed money is needed to start the process, adequate resource mobilization processes for more rollout and long-term sustainability will go a long way in increasing the outcomes of the initiative.”* **Member of Health Professionals Association**

## 5 Recommendations

The summary of recommendations as extracted from the KIIs’ respondents is summarized in Box 1.

BOX 1 **Recommendations.**

1. WHO should continue to engage more countries to have effective response teams to enhance emergency response capacities and facilitate a robust African response team.
2. Member states will need to further engage the trained AVoHC-SURGE responders and have some of them deployed for emergencies within the country and in neighboring countries as practical field experience to sharpen their skills.
3. Member states should collectively develop standard protocols and procedures that all countries will have to follow in the management of AVoHC-SURGE members.
4. The nationally trained AVoHC-SURGE members should be engaged in cascading the training to the lower levels to internalize the training and raise more responders at the lower levels.
5. There is a need to sustain implementation for at least 3 years to measure impact. However, it would be interesting if WHO/AFRO organized a bi-annual or annual review to share experiences.
6. Regular SimEx can be planned to test the responsiveness of the volunteers for emergencies.
7. There is a need to conduct annual performance reviews of the 2-year roadmap at country levels to discuss the performance of the roadmap implementation, barriers, lessons learned, and next steps with stakeholders.

## 6 Conclusion and future action

The implementation of the SURGE flagship initiative in the first year, aimed at producing at least 3,000 emergency responders for WHO/AFRO member states by 2026, has been very eventful. The roll-out process across the first 15 piloted countries has followed uniform but diversely unique approaches, though not without challenges. The foundational roll-out methodology of the initiative and the lessons learned from the whole process, as evident through the outcome and the feedback from the key informants, will serve as the pivot on which the implementation in the remaining countries in the WHO/AFRO will be hinged. The core stakeholders of the initiative will utilize the findings in this documentation study, including the recommendations suggested by the key informants to further improve the quality of the roll-out process in all the countries, and operational solutions will be collaboratively sought and applied to the challenges using holistic and country-specific methodologies.

## Author contributions

IC: Conceptualization, Formal analysis, Project administration, Writing – original draft, Writing – review & editing. FB: Funding acquisition, Resources, Supervision, Validation, Writing – original draft, Writing – review & editing. EA: Data curation, Formal analysis, Validation, Writing – original draft, Writing – review & editing. ED: Validation, Writing – original draft, Writing – review & editing. RN: Validation, Writing – original draft, Writing – review & editing. JO: Supervision, Validation, Writing – original draft, Writing – review & editing. OO: Validation, Writing – original draft, Writing – review & editing. JH: Validation, Writing – original draft, Writing – review & editing. AW: Validation, Writing – original draft, Writing – review & editing. MHD: Validation, Writing – original draft, Writing – review & editing. AK: Validation, Writing – original draft, Writing – review & editing. DK: Validation, Writing – original draft, Writing – review & editing. J-JM: Validation, Writing – original draft, Writing – review & editing. SF: Validation, Writing – original draft, Writing – review & editing. OA: Validation, Writing – original draft, Writing – review & editing. AA: Validation, Writing – original draft, Writing – review & editing. JA: Validation, Writing – original draft, Writing – review & editing. RP: Validation, Writing – original draft, Writing – review & editing. LMM: Validation, Writing – original draft, Writing – review & editing. MD: Validation, Writing – original draft, Writing – review & editing. MK: Validation, Writing – original draft, Writing – review & editing. KD: Validation, Writing – original draft, Writing – review & editing. SC: Validation, Writing – original draft, Writing – review & editing. KM: Validation, Writing – original draft, Writing – review & editing. KB: Validation, Writing – original draft, Writing – review & editing. MO: Validation, Writing – original draft, Writing – review & editing. RW: Validation, Writing – original draft, Writing – review & editing. GJ: Validation, Writing – original draft, Writing – review & editing. GS: Validation, Writing – original draft, Writing – review & editing. GA: Validation, Writing – original draft, Writing – review & editing. PA: Validation, Writing – original draft, Writing – review & editing. MA: Validation, Writing – original draft, Writing – review & editing. ML: Validation, Writing – original draft, Writing – review & editing. HAO: Validation, Writing – original draft, Writing – review & editing. LM: Validation, Writing – original draft, Writing – review & editing. AR: Validation, Writing – original draft, Writing – review & editing. MMB: Validation, Writing – original draft, Writing – review & editing. YK: Validation, Writing – original draft, Writing – review & editing. PL: Validation, Writing – original draft, Writing – review & editing. AMD: Validation, Writing – original draft, Writing – review & editing. GT: Validation, Writing – original draft, Writing – review & editing. MB: Validation, Writing – original draft, Writing – review & editing. AD: Validation, Writing – original draft, Writing – review & editing. BM: Validation, Writing – original draft, Writing – review & editing. AY: Validation, Writing – original draft, Writing – review & editing. OR: Validation, Writing – original draft, Writing – review & editing. LD'k: Validation, Writing – original draft, Writing – review & editing. BB: Validation, Writing – original draft, Writing – review & editing. HO'm: Validation, Writing – original draft, Writing – review & editing. AG: Project administration, Resources, Supervision, Validation, Writing – original draft, Writing – review & editing.
